# Breast Cancer Cells in Microgravity: New Aspects for Cancer Research

**DOI:** 10.3390/ijms21197345

**Published:** 2020-10-05

**Authors:** Mohamed Zakaria Nassef, Daniela Melnik, Sascha Kopp, Jayashree Sahana, Manfred Infanger, Ronald Lützenberg, Borna Relja, Markus Wehland, Daniela Grimm, Marcus Krüger

**Affiliations:** 1 Department of Microgravity and Translational Regenerative Medicine, Clinic for Plastic, Aesthetic and Hand Surgery, Otto von Guericke University, 39106 Magdeburg, Germany; nassefmohamedzakaria@gmail.com (M.Z.N.); daniela.melnik@med.ovgu.de (D.M.); sascha.kopp@med.ovgu.de (S.K.); manfred.infanger@med.ovgu.de (M.I.); ronald.luetzenberg@med.ovgu.de (R.L.); markus.wehland@med.ovgu.de (M.W.); daniela.grimm@med.ovgu.de (D.G.); 2 Research Group “Magdeburger Arbeitsgemeinschaft für Forschung unter Raumfahrt- und Schwerelosigkeitsbedingungen” (MARS), Otto von Guericke University, 39106 Magdeburg, Germany; 3 Department of Biomedicine, Aarhus University, 8000 Aarhus C, Denmark; jaysaha@biomed.au.dk; 4 Experimental Radiology, Department of Radiology and Nuclear Medicine, Otto von Guericke University, 39120 Magdeburg, Germany; borna.relja@med.ovgu.de

**Keywords:** metastasis, proliferation, apoptosis, cell adhesion, cytoskeleton, in vitro 3D tumor model, cancer therapeutic targets

## Abstract

Breast cancer is the leading cause of cancer death in females. The incidence has risen dramatically during recent decades. Dismissed as an “unsolved problem of the last century”, breast cancer still represents a health burden with no effective solution identified so far. Microgravity (µ*g*) research might be an unusual method to combat the disease, but cancer biologists decided to harness the power of µ*g* as an exceptional method to increase efficacy and precision of future breast cancer therapies. Numerous studies have indicated that µ*g* has a great impact on cancer cells; by influencing proliferation, survival, and migration, it shifts breast cancer cells toward a less aggressive phenotype. In addition, through the de novo generation of tumor spheroids, µ*g* research provides a reliable in vitro 3D tumor model for preclinical cancer drug development and to study various processes of cancer progression. In summary, µ*g* has become an important tool in understanding and influencing breast cancer biology.

## 1. Learning from Space

After humans managed to leave Earth’s surface, the era of microgravity (µ*g*) research began. Since the middle of the 20th century, researchers have been investigating how µ*g* affects the human body. Many astronauts and cosmonauts have reported various side effects after long-term space missions in orbit, to the Moon or onboard the International Space Station, Tiangong or the Mir station. They included cardiovascular changes, reduction in bone density, muscle atrophy, and risk of kidney stone formation [1,2,3,4,5,6,7]. Many of these health issues are attributed to the effects of µ*g* on cellular properties. Gravity was near constant during billions of years of evolution on Earth (estimated to be stabilized to 9.8 m/s^2^ after hypothesized mass-changing events such as the Late Heavy Bombardment during Earth formation). Therefore, there is little or no genetic memory in organisms on how to respond to force changes in the low gravity range. Hence, it is likely that terrestrial life adapting to µ*g* will reveal many novel mechanisms that could be helpful in biomedical research [8,9,10].

The relationship between a µ*g* environment and tumorigenesis is a further great concern that has attracted the attention of the academic world [11,12,13,14]. During a stay in space, the immune system of astronauts is affected to varying degrees, resulting in a reduced function of immune cells as well as a reduced ability to control mutated cells [15], among other effects of space radiation. In addition, µ*g* induces alterations in gene expression, signal transduction, proliferation and morphology in a variety of tumor cells by influencing the ‘mechanical tumor microenvironment’ [16,17]. Moreover, thyroid cancer cells were found to develop a more differentiated and less aggressive phonotype when cultured in space [18]. A very important point, however, is that these results were obtained in cancer cell monocultures. For example, µ*g* was also observed to suppress the activity of immune cells, which itself increases the risk of cancer development [16]. To our knowledge, no cancer-bearing organisms has been sent into space as of yet, neither were mice with tumor xenografts studied in orbit. Therefore, further research has to focus on the complex molecular interplay in vivo that determines physiological and biological responses to µ*g*.

When cancer cells grow in µ*g*, they start detaching from their substrate and form three-dimensional (3D) aggregates known as multicellular spheroids (MCS) [19]. Cancer biologists have recognized the importance of µ*g*-generated MCS for cancer research [20]. Due to their close resemblance to the in vivo growth of human tumors, MCS are a promising model to study metastasis and to investigate potential cancer drugs [21,22,23]. In summary, µ*g* can change the growth, migration and invasion ability of cancer cells, and thus displays an interesting tool for cancer research [24,25,26].

This review will summarize the current knowledge about the effects of µ*g* on human breast cancer cells. Breast cancer is the most invasive cancer in women. Tumor heterogeneity is a major problem limiting the efficacy of targeted cancer therapies. Therefore, fighting breast cancer requires to “think outside-the-box”. We address the importance of µ*g* research as a tool that can be used to develop new 3D in vitro model systems for drug screening or even discover novel breast cancer medications.

## 2. Breast Cancer

According to the latest global GLOBOCAN statistics from 2018 [27], breast cancer was responsible for 11.6% of total cancer deaths in both sexes as the second leading cause of cancer death. This year’s cancer statistics by the American Cancer Society shows that breast cancer alone accounts for 30% of all new cancer incidents (and 5% of cancer deaths) in women in the Unites States [28]. Breast cancer represents both a health and an economical burden with a rising number of cases predicted every year. Cancer research is the best approach to fight this malignancy of the disease and to identify novel targets which could be used for the development of new medications. Environmental and lifestyle factors are considered to be the main reasons for developing breast cancer, whereas genetic predisposition accounts for only 10% of cases [29]. Late maternal age at first pregnancy, early menarche, late onset of menopause and lack of breast-feeding account as environmental and lifestyle factors [30]. Other factors such as obesity, physical inactivity and alcohol use were found to increase the risk of developing breast cancer [31]. Mutations in high penetrance genes such as breast cancer 1 (*BRCA1*), breast cancer 2 (*BRCA2*), phosphatase and tensin homolog (*PTEN*), tumor protein P53 (*TP53*), E-cadherin (*CDH1*) and serine/threonine kinase 11 (*STK11*) lead to an increased life-time risk of developing breast cancer up to 80% [32]. Furthermore, mutations in moderate-penetrance genes such as BRCA1 interacting protein C-terminal helicase 1 (*BRIP1*), ataxia telangiectasia mutated (*ATM*), partner and localizer of BRCA2 (*PALB2*), and checkpoint kinase 2 (*CHEK2*) are associated with a two-fold increased risk of developing breast cancer [32]. Women with family history of breast cancer are recommended a genetic evaluation. Next Generation Sequencing is currently the tool of choice for investigation of genetic disorders in breast cancer [29,32].

How breast cancer transformation is initiated is not clear; however, it is divided into seven subtypes based on its molecular characteristics. These seven subtypes are luminal A, luminal B, basal like/triple-negative, human epidermal growth factor receptor (HER)2-enriched, molecular apocrine, claudin-low and normal breast cancer-like [33,34,35,36]. The biological classification of breast cancer improved therapy guidelines and contributed to developing new therapy approaches for each subtype. Moreover, breast cancer is considered hormone-receptor-positive when it possesses estrogen receptor (ER) and/or progesterone receptor (PR), whereas a tumor is considered as triple negative breast cancer when lacking in ER, PR and HER2 expression. Triple negative breast cancer is associated with particularly poor prognosis, as it does not respond to hormonal therapy [29].

Early stage breast cancer is curable in 70-80% of cases, while advanced breast cancer is virtually incurable [29]. Metastasis is the cause of death in almost all breast cancer patients [37]. Breast cancer spreads in the bones, lungs, liver, and rarely to the brain. Metastatic breast cancer patients receive treatment with the aim of relieving symptoms and prolonging life-expectancy [29]. Therefore, it is of utmost importance to diagnose breast cancer in the early stages prior to metastasis. The diagnosis is achieved through biopsies, mammography, ultrasound and breast magnetic resonance imaging. Treatment options include surgery, radiotherapy, chemotherapy and molecular treatment [38,39,40]. The therapy is tailored according to the stage and progression of the disease in the individual patient [29]. To improve the disease outcome, intensive research studies are mandatory to gain a better understanding of the pathogenesis of the disease and to identify novel targets.

Common breast cancer cell lines that have been used in µ*g* research to date are listed in Table 1.

## 3. Breast Cancer Cells Exposed to Microgravity

### 3.1. The Microgravity Environment

The nature of gravity was first described by Sir Isaac Newton over 300 years ago. Gravity is an attractive force, which is always present between two objects that have a mass, most apparent when one mass is very large (like Earth). Near the Earth’s surface, the acceleration of an object toward the ground (9.8 m/s^2^), caused by gravity alone, is called “normal gravity” or 1*g*. The condition of microgravity (µ*g*) is frequently used as a synonym for weightlessness or “zero-*g*”; however, *g*-forces are not actually zero but rather very small. Physically, µ*g* is achieved when the absolute sum of all mass-dependent accelerations is below a noise level of 10^−4^ (per definition, 1 µ*g* = 1 × 10^−6^ g) [42,43]. Real microgravity (r-µ*g*) for more than a few minutes can only be reached in space. Shorter durations of r-µ*g* can be attained in drop towers (<10 s), on parabolic (~22 s) or sounding rocket flights (up to 13 min), whenever an object is in free fall (the properties and r-µ*g* qualities of the different flight platforms are described in Section 3.3). In simulation experiments in laboratories on Earth, the magnitude of the Earth gravity vector cannot be changed. However, its influence can be altered with a simulator resulting in a “functional near-weightlessness” or vector-averaged, “simulated” microgravity (s-µ*g*) [42,43]. Multiple experiments demonstrated that cells and unicellular organisms behave differently under µ*g* conditions (both real and simulated) compared to similar cells exposed to the normal gravity of Earth. Therefore, by removing the effects of gravitational force, it is possible to study the fundamental processes of life down to the cellular level in different organisms [43]. Microgravity is suggested to be a mechanical stressor for adherent cells induced through tensegrity [44]. It is known that mechanical stress influences the aggression and progression of cancer [45]. This is elucidated by changes of cancer cell behavior (e.g., cell survival, proliferation, differentiation) and structure (e.g., cellular shape, alteration in the cytoskeleton) [46].

### 3.2. Cellular Studies in Simulated Microgravity

In recent decades, several devices with different physical concepts have been engineered to simulate µ*g* on Earth. Due to high costs and limited options for experiments in space, s-µ*g* represents a perfect option to test hypotheses or to perform preliminary/preparatory and comparative studies. Moreover, these technologies enable multi-time-point experiments and multiple repetitions.

Various rotational simulators diminish the impact of gravity on biological samples by randomizing the direction of the gravity vector over time, such as 2D/3D clinostats (rotation around one or two axes), the Random Positioning Machine (RPM; a special version of a 3D clinostat, where both frames can operate with different speeds and in different directions) and the NASA-developed Rotating Wall Vessel (RWV) bioreactor, commercially known as Rotary Cell Culture System (RCCS) [43]. Suspending cells in a magnetic field offers another innovative way to study their properties and establish 3D cultures under s-µ*g*, called diamagnetic levitation [47,48].

#### 3.2.1. Growth Behavior

**Figure 1:**
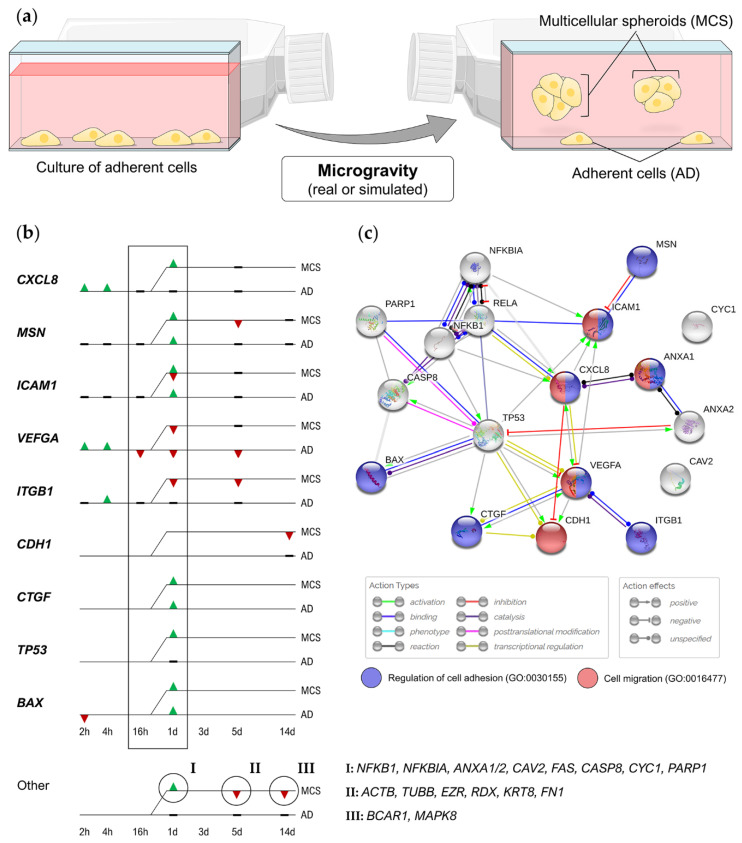
(**a**) Under µ*g* conditions, breast cancer cells grow into two distinct populations, characterized by hugely different morphologies. (**b**) Genes regulated in s-µ*g*-induced multicellular spheroids (MCS) formation of MCF-7 cells [50,51,53]. First MCS were detectable after 24 h of random positioning. ▲, upregulation; ▼, downregulation; (**c**) STRING (Search Tool for the Retrieval of Interacting Genes/Proteins) interaction network of proteins encoded by the regulated genes. Biological processes that are important both in cancer progression and in MCS formation are colorized. Blue, regulation of cell adhesion (Gene Ontology process GO:0030155); red, cell migration Gene Ontology process GO: 0016477). Gene symbols: *ACTB*, β-actin; *ANXA1/2*, annexin 1/2; *BAX*, Bcl-2-associated X protein; *BCAR1*, breast cancer anti-estrogen resistance protein 1; *CASP8*, caspase-8; *CAV2*, caveolin 2; *CDH1*, E-cadherin; *CTGF*, connective tissue growth factor; *CXCL8*, interleukin-8; *CYC1*, cytochrome c1; *EZR*, ezrin; *FAS*, Fas cell surface death receptor; *FN1*, fibronectin; *ICAM1*, intercellular adhesion molecule 1; *IKBKG*, inhibitor of NF-κB kinase regulatory subunit gamma; *ITGB1*, integrin-β1; *KRT8*, cytokeratin; *MSN*, moesin; *NFKB1*, nuclear factor kappa B subunit 1; *NFKBIA*, NF-κB inhibitor A; *PARP1*, poly [ADP-ribose] polymerase 1; *RDX*, radixin; *TP53*, tumor protein p53; *TUBB*, β-tubulin; *VEGFA*, vascular endothelial growth factor A. Parts of the figure are drawn using pictures from Servier Medical Art (https://smart.servier.com), licensed under a Creative Commons Attribution 3.0 Unported License (https://creativecommons.org/licenses/by/3.0).

Like other adherent cell types, breast cancer cells exposed to s-µ*g* grow into two distinct populations, characterized by hugely different morphologies. The first population consists of cells adherent to the substrate roughly preserving their native, spindle profile. The other population comprises small, rounded cells, which are grouped and linked to each other forming MCS floating in the supernatant (Figure 1a) [49]. These MCS represent a valuable 3D in vitro model system to study different aspects of cancer biology and for drug screening (see Section 4). Masiello et al. [49] suggested that during MCS formation in s-µ*g*, non-adherent cells are grouped in discrete clusters establishing tight cell–cell contacts. Thus, their solidity is higher compared to isolated, adherent cells on the RPM. The multiple cell adhesion is suggested to “stabilize” cell shape, by mutually reinforcing their stiffness [49].

Kopp et al. [50] intensely investigated MCF-7 cells for several days on an RPM. After 24 h, the first MCS were observed; MCS continued to increase in number and complexity until day 5. Most interestingly, the MCS showed a duct-like shape resembling human epithelial breast cells in vivo. Using the additional experimental omics data gained from these experiments, a Pathway Studio analysis identified that genes influenced by s-µ*g* are involved in the regulation of cell shape, cell tip formation and membrane-to-membrane docking [50]. Furthermore, the capability of MCF-7 cells to form MCS on the RPM depended on the intracellular distribution of the nuclear factor ‘kappa-light-chain-enhancer’ of activated B-cells (NF-κB) p65 subunit [51]. After 24 h of exposure to s-µ*g*, NF-κB was predominantly localized in the cytoplasm of adherent cells. In contrast, NF-κB was found in the nuclei of MCS cells. The glucocorticoid dexamethasone targeting NF-κB, suppressed MCS formation of MCF-7 cells in a dose-dependent manner [51]. An interaction analysis of 47 investigated genes suggested that heme oxygenase 1 (HMOX-1) and NF-κB variants are activated, when MCS are formed [51]. AU-565 cells indicated enhanced cell repair, modified cell adhesion and phenotypic preservation after 24 h on the RPM [52]. A large proteomics analysis determined the role of the cell junction protein E-cadherin in MCS formation of RPM-exposed cells [53]. E-cadherin was significantly reduced in MCS in comparison to conventionally cultured cells. This decrease in E-cadherin was also observed when MCF-7 cells were exposed to r-µ*g* phases (31 × 22 s) during a parabolic flight [54]. After blocking E-cadherin with antibodies MCS formation was promoted in MCF-7 cells [53]. In contrast, MCS formation was prevented by c-Src (proto-oncogene tyrosine-protein kinase c-Src) inhibition. This finding highlighted the importance of E-cadherin in s-µ*g*-induced MCS formation [53]. Moreover, several studies have detected ubiquitin-like protein ISG15, which is highly expressed in different cancers [53,55,56,57,58]. It is also known as a prognostic marker in human breast cancer [59] and it might play an important role in the stabilization on MCS [53]. By analyzing omics data and semantic searches, Bauer et al. [60] found that the process of linking cells to each other or to the extracellular matrix (ECM) in (s-)µ*g* also includes the sialylation of extracellular domains of adhesion proteins. 

Although these studies offered valuable information about the underlying mechanism of MCS formation in s-µ*g* (Figure 1b,c), the full mechanism remains to be unraveled. However, similar regulation of some genes (*CXCL8*, *VEGFA*, *TP53*, etc.) has been found in various malignant and non-malignant cell types that have been exposed to s-µ*g* [50,61,62,63,64]. This suggests a general mechanism of MCS formation in s-µ*g*, although different culture conditions and time points hamper to compare the existing results directly with each other.

In addition to the common use of rotatory-based s-µ*g*, different breast cancer cells (MCF-7, MDA-MB-231 and MDA-MB-468) also formed vital 3D aggregates when cultured (shear-force-free) under magnetic levitation. Levitated MCS showed high N-cadherin expression and enhanced epidermal growth factor receptor activity [65,66]. However, levitation research on breast cancer is just beginning and further studies are necessary to compare the effects of magnetic levitation with that of rotating bioreactors.

#### 3.2.2. Cytoskeleton Architecture

When human cells are exposed to µ*g* (both simulated and real; see also Section 3.3.1) the cytoskeleton undergoes a dramatic reorganization, resulting in the activation of various genes which can trigger different biochemical pathways [67,68,69]. Hereby, the complex interplay between tensional forces and the cytoskeleton architecture modulates several essential cell functions such as proliferation, differentiation, apoptosis or ECM remodeling. The cytoskeleton is therefore often referred to as a gravity sensor [70].

Particularly for tumor cells, it has been reported that actin arrangement and remodeling can be related to their metastatic potential [71]. Invasive MDA-MB-231 cells showed a large rearrangement of F-actin after 24 h of RPM exposure. These rearrangements were almost stable and could been observed even after 72 h on the RPM [49]. Interestingly, the cytoskeleton changes greatly differed between the two cell populations: in adherent MDA-MB-231 cells, the complex cytosolic actin network disappeared, and actin was mostly localized on the cell border. In MCS cells, the F-actin network was completely disrupted [49]. Li et al. [72] described that non-invasive MCF-7 cells exposed to a clinostat did not display their typical radial actin array during 7 days of observation. These cells seemed to re-adapt to the s-µ*g* environment as part of the newly formed actin structures (e.g., lamellipodia) were reversed after 48 h. This is in accordance with the findings of Chiotaki et al. [73] who described that MDA-MB-231 cells appear to require a stable actin cytoskeleton to maintain a consistent nuclear periphery, while this is not the case for MCF-7 cells. Kopp et al. [50] further reported that both MCF-7 cell populations showed an accumulation of F-actin at the cell borders in addition to pronounced holes in the F-actin network after 5 days on the RPM. Stress fibers were only visible in adherent cells, indicating a higher mechanical load on these cells during random positioning. Molecular analyses revealed that *ACTB* and *TUBB* genes, as well as mRNAs of ezrin (*EZR*) and radixin (*RDX*) linking actin to the plasma membrane, were downregulated in the MCS cells [50]. Similar effects were observed when AU565 breast adenocarcinoma cells (which overexpress HER2/neu) were exposed to the RPM: actin filaments accumulated at the cell border after 5 days. At the same time, gene expression of transforming protein RhoA (*RHOA*) was upregulated [74]. The involvement of RhoA in s-µ*g*-induced actin (re-)organization seems to be cell type-specific, since the *RHOA* mRNA levels were unaltered in RPM-exposed MCF-7 cells [50]. 

Microtubules were disrupted in adherent MCF-7 cells after 4 h of clinorotation, but were partly reestablished by 48 h [72]. In adherent MDA-MB-231 cells grown on the RPM for 24–72 h microtubules were disorganized, with a more evident thickening in perinuclear position, whereas in MCS cells, the tubulin network was completely disrupted showing a slight diffuse fluorescence throughout the entire cytoplasm [49]. Considering that microtubule-associated tumor suppressors (such as breast cancer 1, BRCA1) can have major impact on cancer aggressiveness and clinical outcome [75], microtubule rearrangement through s-µ*g* can indeed be a tool to influence the latter.

The intermediate filament vimentin is selectively expressed in aggressive breast cancer cell lines such as MDA-MB-231 correlating with increased migration and invasion abilities of these cancer cells [76]. After exposing MDA-MB-231 cells to the RPM for 3 days, the vimentin network was disrupted in both adherent and MCS cells. Vimentin formed dense aggregates close to the nucleus [49] and thereby maybe influencing nuclear shape, mechanics and chromatin organization [77]. In AU565 cells the VIM gene was upregulated after 5 days on the RPM [74].

In summary, there are many indications that mechanical stimuli created by s-µ*g*, cytoskeletal structure, and cellular behavior are tightly linked. Studying the mechanisms that underlie these processes will contribute to identify key molecular targets guiding to new therapeutics based on the tumor mechanobiology.

#### 3.2.3. Cell Cycle and Proliferation

Influence of s-µ*g* on the cell cycle was found in MCF-7 cultured on a clinostat, as cells in G2/M phase were significantly increased after 24 h and 48 h of clinorotation [78]. Chen et al. [79] investigated MCF-7 and MDA-MB-231 cells for 5 days on an RCCS. Simulated µ*g* did not affect the overall growth rate of these cancer cells, but the authors reported a significant accumulation of adherent cells in the S phase of the cell cycle. The induction of cell cycle arrest on the RCCS can most likely be attributed to the inhibition of cyclin D1 [79], a master regulator of the cell cycle that is required for re-entry into G1-phase after quiescence. Cyclin D1 overexpression is reported in >50% of human breast cancers and dysregulation of cyclin D1 expression or function contributes to altered cell cycle control during breast cancer development [80]. Masiello et al. [49] analyzed both cell populations of RPM-treated MDA-MB-231 cells in detail. A slight increase in the S phase distribution of adherent cells could be observed. However, non-adherent cells showed an impressive decrease in the S phase distribution paired with an accumulation of cells in the G2/M phase both after 24 and 72 h, demonstrating a persistent inhibition of cell growth. Furthermore, a significant decrease in cyclin D1 levels was recorded in MCS, whereas cyclin D1 levels were higher in adherent cells [49]. Considering that proper functioning of the mitotic process requires a correct arrangement of the tubulin meshwork [81], disorganization of tubulin microfilaments in non-adherent cells (either directly caused by s-µ*g* or by s-µ*g*-induced cyclin D1 repression) may help in elucidating the cell cycle arrest. Though, when Zheng et al. [82] cultured MCF-7 MCS encapsulated in 3D collagen-alginate hydrogels on an RCCS, neither the cytoskeleton distribution nor the assembly of mitotic spindle were altered by rotary culture. Nevertheless, cells in S and G2/M phase were significantly increased compared to the control group. This study also indicated that the RCCS promotes the proliferation of encapsulated MCF-7 MCS cells by inducing the ERK1/2 pathway [82].

#### 3.2.4. Apoptosis 

Qian et al. [78] reported that clinorotation induced apoptosis in MCF-7 cells after 72 h. However, they did not conduct a molecular analysis to explain their observation. Studies using the RPM often revealed contrasting behaviors of MCF-7 cells: an increase in apoptosis after 24 h [51], but no obvious change in apoptosis-related proteins after 48 h [83]. The investigations by Masiello et al. [49] may explain the different results through the presence of different cell populations. The authors found that the apoptotic process was particularly enhanced in non-adherent MDA-MB-231 cells on the RPM (increase in pro-apoptotic effectors after 24 and 72 h), in contrast to adherent cells which displayed only minor changes in apoptosis. Similarly, Nassef and coauthors [84] detected no apoptosis in adherent MDA-MB-231 cells after short-term exposure to the RPM, but the cells responded to s-µ*g* with transcriptional downregulation of both anti-apoptotic annexin A2 and pro-apoptotic Bax (Bcl-2-associated X protein) after 2 h. Bcl-2 (B-cell lymphoma 2) plays an important role in tumor development by regulating the endogenous apoptosis pathway which can inhibit apoptosis [85]. This way, it can counteract the pro-apoptotic stress occurring during tumorigenesis. Recently, Jiang et al. [86] described that apoptosis of MDA-MB-231 cells was promoted by RCCS culture (7 days) by decreased expression of the anti-apoptotic Bcl-2 as well as increased expression of the pro-apoptotic Fas protein, further accompanied by the appearance of a large number of lysosomal and vacuolar structures.

This leads to assume that a s-µ*g* environment can induce apoptotic processes in cancer cells including breast cancer cells. Their regulation in MDA-MB-231 cells comprises an interplay between different cell signaling pathways, mainly involving the activation of p-ERK and p-AKT expression. Both p-AKT and p-ERK were significantly reduced in suspended (apoptosis-sensitive) cells but increased in the adherent (apoptosis-resistant) cell population exposed to an RPM [49]. Unravelling the underlying molecular mechanisms of apoptosis control might indeed bear a potential for new targets in cancer drug discovery. However, some overly enthusiastic reports about killing breast cancer cells in s-µ*g* have to be evaluated critically [87].

#### 3.2.5. Cell Adhesion and Migration Ability

Microgravity causes disorganization in the focal adhesions (FAs) of adherently growing cancer cells, which is most likely connected to MCS formation (suspension cell population) and crucial for cell migration including metastasis. Creating cell movement requires the maturation of FAs, which serve as traction points to propel the cell forward [88]. An important step during the maturation process is binding and clustering of integrins to establish a physical and functional link between the cytoskeleton and the ECM [89]. Li et al. [72] detected that the number of FAs generated in s-µ*g* was reduced and these FAs were less mature (clustered) than those established under normal gravity conditions. In addition, s-µ*g* attenuated the expression of integrin-β1 and integrin-β4 and affected adhesion signaling by decreasing FAK, PYK2, and ILK kinase activity in MCF-7 cells [72]. Chen [90] later proposed that both decreased integrin expression and downregulated FA kinase activities are key processes in suppression of breast cancer cell migration under s-µ*g*. Most recently, Shi et al. [91] performed experiments with MCF-7 cells on an RWV and reported that EMT transcription factors (Snail, Twist, and ZEB1) are also involved in the alteration of cell adhesion properties under s-µ*g*. This might be a possible response of breast cancer cells to stress changes, in regulating the expressions of adhesion proteins and thus adapting their adhesion state to the altered mechanical (s-µ*g*) environment [91].

Cell migration is a fundamental process in the development of multicellular organisms [92]. Even in adult organisms, the migration of specialized cells is still essential, e.g., for proper immune response, wound repair, and tissue homeostasis. However, during metastasis, this process converts to the characteristically malignant behavior of tumor cells. The s-µ*g* environment is known to change the migration and invasion ability of various tumor cells [93,94]. For MCF-7 cells, Qian et al. [78] found that the invasive ability decreased after clinostat exposure together with a reduced expression of matrix-metallopeptidases (MMP-2, MMP-9) and vinculin. A transwell migration assay showed that the migration of MDA-MB-231 cells was also significantly reduced after 72 h on an RCCS [86]. The authors concluded that the decrease in MMP-9 expression in MDA-MB-231 cells might be closely related to a decrease in migration ability in s-µ*g*.

#### 3.2.6. Matrix Composition and Stiffening

The development of tumor tissue is often associated with ECM accumulation after the transition from the avascular growth phase of cancer cells [95]. As a result, the tumor tissue is often much stiffer than the host tissue [96]. Elastic modules of human breast cancer lesions were measured one order of magnitude higher (10–42 kPa) than the typical elastic modules of normal breast tissue (3.25 kPa) [97]. The cultivation of MCF-7 cells on the RPM induced changes in the ECM, including laminin and fibronectin. In particular, fibronectin was downregulated after 5 days in adherent cells cultured on the RPM, whereas laminin was reduced in MCS cells indicating a reduction of ECM stiffness and tumorigenicity in MCF-7 cells after s-µ*g* treatment [50]. Data gained from breast cancer cells in conventional 3D suspension culture suggest that increased cellular fibronectin could promote the initial attachment of cancer cells to secondary sites during metastasis [98]. However, there have been no comparative studies in shear force-free r-µ*g* so far to elucidate (possible) effects of mechanical stress on the ECM composition.

#### 3.2.7. Cancer Cell Metabolism

In an early study, Coinu et al. [83] demonstrated that the metabolic activity of MCF-7 cells was attenuated after a 2-day exposure to an RPM. They found reductions in glucose and methionine uptake and decreased thymidine incorporation as an effect of s-µ*g*. A gene array analysis of MCF-7 cells after RPM-treatment revealed a > 2-fold change of only 23 genes and among them several were coding for proteins that are affected by oxygen levels or regulate glycolysis [51]. Comparing ERα positive MCF-7 and ERα negative MDA-MB-231 cells on an RCCS, Zheng et al. [99] reported an important role in protecting cells from oxidative stress damage under s-µ*g*. Later studies investigating both cell lines on the RCCS revealed changes in energy metabolism as well as increases in intracellular lactic acid and lactate dehydrogenase activity. Chen et al. [79] reported that several metabolic pathways were affected after 5 days of s-µ*g* including a number of pathways involved in glycometabolism (e.g., glycolysis, Krebs cycle, pentose phosphate pathway, and glycerol-phosphate and malate-aspartate shuttles). The authors suggested that different types of cancer cells can reprogram their metabolism to fulfill particular demands after long-term exposure to s-µ*g*, including processes involved in cell proliferation, metastasis, immunological escape and survival, thus shifting their aggressive phenotype [79]. Metabolic reprogramming is regarded as a characteristic of tumor cells [100]. Van der Heiden et al. [101] explained that the elevated expression of glycolytic enzymes in cancer cells, the molecular basis of the Warburg effect, is connected to the increased stability of hypoxia inducible factor 1α (HIF1α), which presents the master transcriptional regulator of glycolysis. Although the exact molecular mechanisms are still unknown, data have shown that glycolysis can not only be induced by hypoxia (and HIF1α), but also by high levels of adrenomedullin under s-µ*g* conditions [79]. This finding represents a novel mechanism of glycolysis activation and further aspect to understand the Warburg effect in MCF-7 and MDA-MB-231 cells.

#### 3.2.8. Cancer Treatment

Recently, Hekmat et al. [102] demonstrated that s-µ*g* can affect the antiproliferative effect of TiO_2_ nanoparticles on MDA-MB-231 cells. After 48 h of clinorotation in the presence of the sterilized nanoparticles, cell viability decreased noticeably without changing morphology. So far this was the first study investigating the influence of s-µ*g* on the effects of breast cancer treatment. However, results from leukemic cells have already indicated that s-µ*g* is able to modulate the cancer cell response to chemotherapy [103]. Authors proposed that (s-)µ*g* can be a powerful additional treatment for cancer cells and an immunomodulatory tool for the development of new immunotherapies, offering to open up new horizons to novel strategies for breast cancer therapy [102,103].

### 3.3. Cellular Studies in Real Microgravity

In addition to drop towers, which enable a very short r-µ*g* phase of a few seconds, there are different orbital and sub-orbital platforms to perform experiments in r-µ*g*: Parabolic flights are frequently offered and the easiest to participate in and therefore considered the platform of choice for researchers to gain access to r-µ*g* experiments. However, repeating r-µ*g*-phases during the flight are flanked by hypergravity phases and interrupted by 1*g*-phases, making it difficult to identify the effects solely attributed to r-µ*g* [104]. In addition, airplane vibration is often transferred onto the experiment and represents a further mechanical stimulus for the cells. Sounding rocket flights provide a longer, vibration-free r-µ*g*-phase (in Europe, MAXUS sounding rocket flights with r-µ*g* times of up to 13 min are achievable so far), after a phase of strong hypergravity (peak acceleration ~13*g*, depending on the rocket/motor combination) during the launch [105]. For long-term studies in r-µ*g*, remote-controlled experiments on satellites (e.g., Bion, Photon) or automated experiments onboard the ISS are currently available alternatives.

#### 3.3.1. Cell Ultrastructure / Cytoskeleton

**Figure 2:**
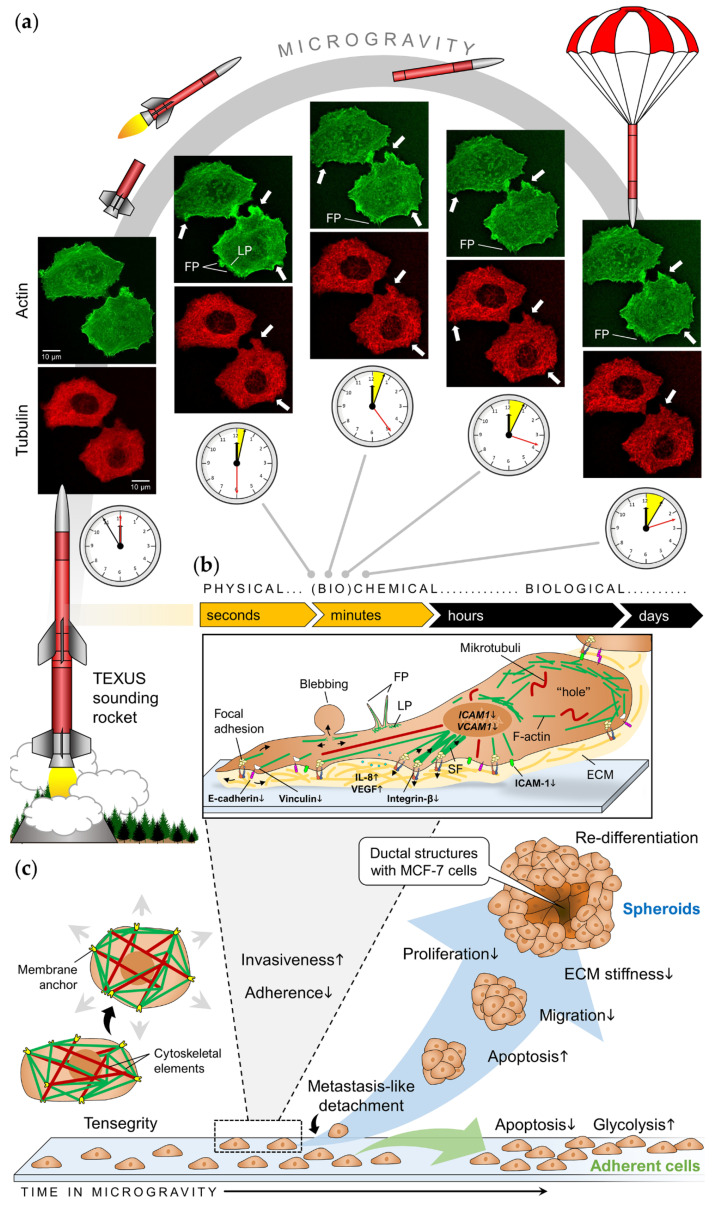
(**a**) Time course images of MCF-7 cells taken on board of the TEXUS-54 sounding rocket during microgravity in comparison to a control image taken 5 min prior to launch. The arrows indicate changes in F-actin (green channel) or α-tubulin (red channel). F-actin cytoskeleton shows appearance of filopodia- and lamellipodia-like structures, accumulation of F-actin, while α-tubulin shows a loose structure and rearrangement of the cytoskeleton. (**b**) Subsequent cellular alterations of adherently growing breast cancer cells after exposure to r-µ*g* (modified from [54]). F-actin is shown as green lines, the extracellular matrix (ECM) in yellow. The effects observed on parabolic and sounding rocket flights were observed in a time-range from a few seconds until minutes. (**c**) Downstream key processes in both cell populations of human breast cancer cells during long-term exposure to s-µ*g*. FP, filopodia; LP, lamellipodia; SF, stress fibers.

The first ultrastructural studies with MCF-7 cells in r-µ*g* were executed by Vassy et al. [106,107] during the Photon 12 mission (CNES, 1999). The cells were fixed after 1.5, 22, and 48 h after launching and responded to r-µ*g* by prolonging cycling and mitosis. Moreover, the cells exhibited a loose perinuclear cytokeratin network and chromatin structure, reduced cell proliferation and altered microtubule structure. Life-cell imaging during the TEXUS-54 sounding rocket flight (DLR, 2018) confirmed a fast rearrangement of the tubulin network in MCF-7 cells (within 150 s in r-µ*g*), together with a disturbance of actin bundles and appearance of cortical filopodia- and lamellipodia-like structures in the actin cytoskeleton (Figure 2a) [54]. An emerging role for microtubules in metastasis has long been suggested and there is increasing interest in the crosstalk between tubulin interacting proteins and actin [108]. The actin cytoskeleton is involved in many processes that are essential for normal physiology [109], but it is known that the actin cytoskeletal architecture can suppress the invasion of ER^+^ breast cancer cells [110]. Some years ago, Mouneimne and coworkers [111] analyzed cancerous tissue and cell samples and found that ER can suppress the invasiveness of breast cancer cells by regulating the expression of actin-binding proteins which affect the formation of actin-driven cell membrane protrusions via suppressive cortical actin bundles. Especially the Ena/VASP-like (EVL) protein has been characterized to promote the generation of these bundles as well as the formation of stress fibers leading to transient cell stiffening. This stability is important during premalignant tumor growth [112]. Even if EVL has not been investigated in µ*g* yet, µ*g* certainly influences the cytoskeletal architecture of breast cancer cells in a way that can impact tumor cell migration and proliferation, and therefore provide an important tool to study metastasis or to develop novel therapeutics against aggressive and metastatic disease [108].

#### 3.3.2. Cell Adhesion and Invasiveness

Changes in gene expression occur very quickly after breast cancer cells are exposed to altered gravity conditions. Both cell lines, MCF-7 and MDA-MB-231, responded to short-term r-µ*g* by modifications in the expression of invasiveness factors and adherence genes (Figure 2b) [54,84]. Enhanced levels of VEGF-A and interleukin-8 together with a down-regulation of vinculin, integrin-β1 and E-cadherin indicated the shift to a more invasive function of MCF-7 cells exposed to short-term r-µ*g* during parabolic flight maneuvers. In MDA-MB-231 cells, the adhesion molecules ICAM-1 (intercellular adhesion molecule 1) and VCAM-1 (vascular cell adhesion molecule 1) were increased after 31 r-µ*g* phases of a parabolic flight. In contrast, there was no expression change of ICAM-1 and VCAM-1 in MDA-MB-231 cells cultured on the RPM for 2 h (total duration of the parabolic flight) [84]. These results may indirectly reflect different biological effects caused by r- and s-µ*g*. The effect may also depend on the duration of r-µ*g* exposure. Thyroid cancer cells showed signs of increased invasiveness after being exposed to short-term r-µ*g* on a parabolic flight, but developed a less aggressive phenotype after being cultured in space for 10 days [18]. However, to our knowledge, no long-term r-µ*g* experiments have been performed on human breast cancer cells as of yet. Table 2 provides an overview of all previous studies investigating breast cancer cells exposed to µ*g*.

## 4. Microgravity-Generated 3D Breast Cancer Models

### 4.1. Homogenous Tumor Spheroids

Multicellular architecture is one of the defining characteristics of breast cancer. Unfortunately, most in vitro tumor models fail to reconstruct tumor architecture or are unable to predict in vivo cellular responses to therapeutics accurately [113]. However, this architecture is known to drive the tumor progression through cell/cell- and cell/matrix-contacts, forced depolarization and reduced tensional homeostasis [114]. Moreover, it leads to the formation of a complex microenvironment characterized by metabolic, catabolic and oxygen gradients, which can only be resembled in 3D cell culture [115].

**Figure 3:**
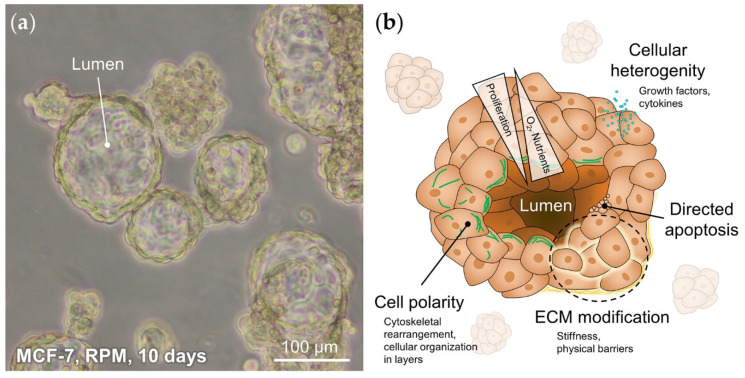
(**a**) Glandular MCF-7 spheroids grown in s-µ*g* for 10 days. (**b**) Schematic diagram and special features of a glandular breast cancer spheroid.

Studies have repeatedly shown that various types of human cells form scaffold-free 3D aggregates after exposure to s-µ*g* or r-µ*g* [19,64,115,116,117,118]. This behavior has also been observed for breast cancer cells already after the first 24 h. An unexpected finding was the formation of glandular MCF-7 spheroids after at least 5 days on the RPM (Figure 2c and Figure 3a), containing duct-like structures with unicellular borders and polarized cells [50]. To form these structures, cells must adapt various processes such as cell/cell- and cell/matrix communication, directed apoptosis, differentiation, and polarization (Figure 3b). Especially for cell polarity, cytoskeletal filaments and associated proteins, that are influenced by µ*g*, may play a significant role [119].

After generation in (s-)µ*g*, these MCS are suitable for studies with chemotherapy, gene therapy, cell- and antibody-based immunotherapy, etc., under normal laboratory conditions [120]. The special 3D architecture of MCS might facilitate the penetration and action of cancer drugs more closely mimicking the situation of a breast tumor in the human body. This way, MCS could help to investigate and understand cell aggregation and to improve the penetration and action of drugs for cancer therapy. However, due to avascular growth, the size of homogenous tumor MCS is still limited.

### 4.2. Heterogeneous Breast Tumor Models

Tumor heterogeneity is one of the hallmarks of breast cancer malignancy. Breast tumors often contain morphologically and molecularly different cell populations (intratumor heterogeneity), resulting in different behavior, presentation, and prognosis [121]. In order to meet these requirements for a 3D tumor model, Vamvakidou et al. [122] generated a coculture-based 3D tumor model out of MDA-MB-231, MCF-7, and ZR-751 cells. The different cell lines were cocultured in an RWV and formed a large number of heterogeneous aggregates. The most important feature was the temporal-spatial organization of the MCS, including the presence of central necrotic areas and higher levels of cell proliferation at the MCS periphery [122].

In a bioreactor experiment that was carried out in 1998 during the last Mir increment, breast cancer cells were co-cultured with a fibroblast layer, made up of angiogenic cells. This was the very first attempt to investigate vascularization of a solid tumor in r-µ*g*. In the tissue that returned from space half a year later, vascularization was initiated [123]. Some years later, Kaur et al. [124] used s-µ*g* to generate a 3D breast cancer model, which consisted of cancer cells (UACC-893, BT-20, or MDA-MB-453) and fibroblasts. Co-cultures resulted in the generation of ‘histoids’ with cancer cells invading fibroblast spheroids.

In another approach, different breast cancer cell lines (MCF-7, MDA-MB-231, and MDA-MB-468) were co-cultured with fibroblasts and then magnetically levitated. The data of Jaganathan and coworkers [125] indicated that the formed 3D aggregates were advantageous due to the ability to: (1) form large MCS within 24 h (millimeter in diameter); (2) resemble tumor cell architecture and density; (3) accurately mimic properties of the in vivo tumor microenvironment; and (4) test drug efficiency in an in vitro model that is comparable to in vivo breast tumors [125].

Heterogenous spheroids grown in s-µ*g* provide a high-throughput in vitro model that could help to understand early stages of cancer development. Furthermore, they can be used as a drug testing or a drug delivery system [122].

### 4.3. Advantages of Microgravity-Generated Spheroids

Reproducibility and physiological relevance are two essential features for in vitro test systems used in drug development [126]. MCS are conventionally generated using bioprinting or with several culture methods such as liquid overlay, hanging drop and hydrogel-based cultures [127,128,129]. One of the important elements required in most techniques for MCS generation is the presence of a synthetic or naturally-derived scaffold that should mimic the ECM [130,131]. However, the addition of an artificial scaffold may impede cell growth and affect cell interactions. In addition, the growing in vivo environment cannot be reconstructed by the static concentration of the scaffold [132]. In µ*g*, cells promote 3D self-assembly without using an artificial scaffold and without the need of external surface for support enabling the simulation of changing in vivo environment. Another advantage of studying cells in µ*g*, sedimentation and buoyancy demonstrated to be neglectable in µ*g* so that it is possible to observe the minimal alterations in biological and physical systems [19]. Microgravity-generated MCS with other cell types do not develop necrosis even when cultured for several weeks in s-µ*g* and therefore are suitable for long-term experiments [50,133]. In comparison to other current technologies the s-µ*g*-based method reproducibly provides a large number of MCS in a short time. Furthermore, due to the ability to reach large diameters (up to 0.5 mm) in a short time, the s-µ*g* MCS model exhibits the condition of in vivo lesions more accurate, including the occurrence of hypoxic regions inside the 3D aggregates. Long-term studies over months need to be performed to study the appearance of necrosis in the MCS.

## 5. Summary and Perspectives

Previous studies indicate that µ*g*, both real and simulated, has a significant impact on breast cancer cells. Changes in cell proliferation, survival, apoptosis and growth behavior had been observed both in triple-negative MDA-MB-231 cells and well-differentiated MCF-7 cells exposed to the s-µ*g* [49,50,51,84]. Gravity-sensitive signaling is required for the very early (24 h) formation of MCS during random positioning. Molecular analyses revealed that inflammatory cytokines (IL-8), NF-κB p65, cell adhesion molecules (ICAM-1, VCAM-1), and growth factors (TGF-β, VEGF-A) seem to be involved in MCS formation in breast cancer cells on the RPM [84]. Particularly, inflammatory cytokines are key players in tumor initiation, promotion, angiogenesis, and metastasis regulating both the induction and the protection in breast cancer [134]. Using the liquid overlay technique, it was shown that interleukins IL-8 and IL-6 can directly induce MCS formation of thyroid cancer cells grown on agarose at 1*g* [129]. IL-8 was upregulated early in MCF-7 cells exposed to short-term r-µ*g* during parabolic flight maneuvers and might start the signaling process for MCS formation [54]. The regulation of IL-6 has not been investigated in breast cancer cells exposed to µ*g* so far, but was it was found elevated in other cancer cells under these conditions [116,135]. In addition, MCF-7 cells overexpressing IL-6 initiated EMT and exhibited a more invasive phenotype [136]. Since MCS formation represents a valuable in vitro metastasis model, targeting cytokines and/or NF-κB might be a promising approach for breast cancer treatment. Genetic alterations related to the cytoskeleton, cell adhesion, ECM and cytokines have been detected with other malignant and non-malignant human cells in a similar way. This may hint towards an overarching mechanism of MCS formation in cancer cells that has to be validated in future studies.

Today’s technologies are constantly providing new insights into the development and biology of breast cancer. The quest for treatment options is advancing just as quickly. So, what is the significance of µ*g* research? On one hand, epidemiological studies have shown an increased breast cancer incidence in female commercial flight attendants (which are exposed to similar occupational conditions as astronauts thought to raise the risk of developing breast cancer without considering further risk factors for cancer such as ionizing radiation) [137], on the other hand, the aggressiveness of cancer cells seems to be reduced in vitro after s-µ*g* exposure [18]. To explain this discrepancy, it is worth mentioning that different biological systems were investigated in these studies and effects found in vitro do not always reflect the complex situation in vivo which also comprises cell/cell interactions and the crosstalk between different body systems in space (disrupted melatonin homeostasis, changes in immune function, etc.). Sending patients into space will certainly not be the cure for breast cancer. Strictly speaking, a lot of research focused on cancer development and progression in space is still needed, especially within the context of planned long-term missions to the moon and Mars. However, µ*g* research offers a unique in vitro tool to investigate biomechanical effects in cancer biology. Microgravity is not directly an option for cancer treatment but supports cancer research in two ways: First, µ*g* research provides a reliable in vitro 3D tumor model for preclinical cancer drug development; second, it has unraveled some remarkably interesting aspects of cancer cell biology. These can be used in other approaches to increase efficacy and precision of future cancer therapies and thus enhance survivability and quality of life for breast cancer patients.

## Figures and Tables

**Table 1:** Features of different breast cancer cell lines used in microgravity studies (modified from [41]).

Cell Line	Tumor	Subtype	ER	PR	HER2	Characteristic Expression
AU565	AC	HER2 enriched	‒	‒	+	EGFR, HER3, HER4, p53
BT-20	IDC	Triple-negative	‒	‒	‒	Wnt-3, Wnt-7B
MCF-7	IDC	Luminal A	+	+	‒	Wnt-7B, IGFBP2, -4, -5
MDA-MB-231	IDC	Claudin-low	‒	‒	‒	EGFR, TGFα, Wnt-7B
MDA-MB-468	AC	Triple-negative	‒	‒	‒	EGFR, TGFα

AC, adenocarcinoma; EGFR, epidermal growth factor receptor; HER, human epidermal growth factor receptor; IDC, invasive ductal carcinoma; IGFBP, insulin-like growth factor-binding protein; TGFα, transforming growth factor alpha.

**Table 2:** Studies reporting the effects of microgravity on human breast cancer cells.

Cell Line	µ*g* Condition (Duration)	Findings	Ref.
MCF-7	Clinostat (24–72 h)	Alterations of cell invasion, migration, adhesion, cell cycle and vinculin expression	[78]
	RPM (2 h–5 d)	After 24 h: compact spheroids; after 5 d: duct-like spheroids; downregulation of *ACTB*, *TUBB*, *EZR*, *RDX*, *FN1*, *VEGFA*, *FLK1*, *CASP3*, *CASP9*, *PRKCA*	[50]
	RPM (24 h)	Translocation of RelA into the nucleus, upregulation of *ANXA1*, *ANXA2*, *CTGF*, *CAV2*, *ICAM1*, *FAS*, *CASP8*, *BAX*, *TP53*, *CYC1*, *PARP1*	[51]
	RPM (48 h)	Reduction of glucose uptake, methionine uptake/incorporation, thymidine incorporation, proliferation, and metabolic machinery	[83]
	RPM (14 d)	Downregulation of *CDH1* and E-cadherin protein in MCS	[53]
	PFC (31 × 22 s)	Upregulation of *KRT8*, *RDX*, *TIMP1*, *CXCL8*; downregulation of *VCL* and E-cadherin protein	[54]
	Sounding rocket (6 min)	Disturbance of F-actin bundles, appearance of filopodia- and lamellipodia-like structures; rearrangement of the tubulin network.	[54]
	Satellite (1.5–24 h)	Prolonged cycling/mitosis, loose perinuclear cytokeratin network and chromatin structure, reduced cell proliferation; altered microtubule structure	[106,107]
MDA-MB-231	RPM (24–72 h)	Reorganized cytoskeleton; alterations in ERK, AKT and survivin signaling pathways	[49]
	RPM (2 h)	Downregulation of *ANXA2*, *BAX*	[84]
	RCCS (7 d)	Impaired cell cycle and ultrastructure, increased apoptosis, decreased migration ability and decreased expression of *BCL2*, *MMP9*	[86]
	PFC (31 × 22 s)	Upregulation of *NFKB1*, *RELA*, *ERK1*, *ICAM1*, *NFKBIA*, *NFKBIB*, *FAK1*, *SPP1*, *CD44*; reduced levels of RelA, osteopontin, increased levels of ICAM-1, VCAM-1; changes in cell adhesion	[84]
AU565	RPM (24 h)	Upregulation of *BRCA1*, *VCAM1*; downregulation of *KRAS*, *VIM*; enhanced cell repair, modified cell adhesion	[52]
	RPM (5 d)	Upregulation of *VIM*, *RHOA*, *BRCA1*, *MAPK, ERBB2*; downregulation of *VEGFA*	[74]

MCS, multicellular spheroid; PFC, parabolic flight campaign; RCCS, Rotary Cell Culture System; RPM, Random Positioning Machine. AKT, protein kinase B; ERK, extracellular-signal regulated kinase; RelA, transcription factor p65. Gene symbols: *ACTB*, β-actin; *ANXA*, annexin; *BAX,* Bcl-2-associated X protein; *BCL2*, B-cell lymphoma 2; *BRCA1*, breast cancer 1, early-onset; *CASP*, caspase; *CAV2*, caveolin-2; *CD44*, cluster of differentiation 44; *CDH1*, E-cadherin; *CTGF*, connective tissue growth factor; *CXCL8*, interleukin-8; *CYC1*, cytochrome c1; *ERBB2*, v-erb-b2 erythroblastic leukemia viral oncogene homolog 2 (=HER2); *ERK1*, extracellular-signal regulated kinase 1; *EZR*, ezrin; *FAK1*, focal adhesion kinase 1; *FAS*, Fas cell surface death receptor; *FN1*, fibronectin; *FLK1*, fetal liver kinase 1, *ICAM1*, intercellular adhesion molecule 1; *KRAS*, Kirsten rat sarcoma; *KRT8*, cytokeratin; *MAPK*, mitogen-activated protein kinase; *MMP9*, matrix metallopeptidase 9; *NFKB1*, nuclear factor kappa B subunit 1; *NFKBIA*, NF-κB inhibitor alpha; *NFKBIB*, NF-κB inhibitor beta; *PARP1*, poly [ADP-ribose] polymerase 1; *PRKCA*, protein kinase C alpha; *RDX*, radixin; *RELA*, transcription factor p65; *RHOA*, Ras homolog family member A; *SPP1*, osteopontin; *TIMP1*, tissue inhibitor of metalloproteinases; *TP53*, tumor protein p53; *TUBB*, β-tubulin; *VCAM1*, vascular cell adhesion molecule 1; *VCL*, vinculin; *VEGFA*, vascular endothelial growth factor A; *VIM*, vimentin.

## Abbreviations

2D	Two-dimensional
3D	Three-dimensional
ECM	Extracellular matrix
ER	Estrogen receptor
HER2	Human epidermal growth factor receptor
µ*g*	Microgravity (r-µ*g*, real microgravity; s-µ*g*, simulated microgravity)
MCS	Multicellular spheroids
RCCS	Rotary Cell Culture System
RPM	Random Positioning Machine
RWV	Rotating Wall Vessel
PR	Progesterone receptor
